# Individual differences in proactive interference in rats (*Rattus Norvegicus*)

**DOI:** 10.3758/s13423-021-01998-7

**Published:** 2021-09-24

**Authors:** Elias Tsakanikos, Phil Reed

**Affiliations:** 1 Roehampton University, London, UK; 2 Department of Psychology, Swansea University, Singleton Park, Swansea, SA2 8PP UK

**Keywords:** Proactive interference, Behavioral types, Individual differences, Latent inhibition

## Abstract

Individual differences in behaviors are seen across many species, and investigations have focused on traits linked to aggression, risk taking, emotionality, coping styles, and differences in cognitive systems. The current study investigated whether there were individual differences in proactive interference tasks in rats (Rattus Norvegicus), and tested hypotheses suggesting that these tasks should load onto a single factor and there should be clusters of rats who perform well or poorly on these tasks. The performance of 39 rats was tested across three learning tasks that all involved disengagement from an irrelevant previously learned stimulus to a relevant stimulus: latent inhibition (LI), partial reinforcement extinction effect (PREE), and reversal learning (RL). An exploratory factor analysis revealed the existence of one factor underlying performance. A cluster analysis revealed the existence of sets of rats displaying either weak LI and strong PREE and RL effects, or vice versa. These findings suggest that proactive interference may be based on a single underlying psychological system in rats.

**Supplementary Information:**

The online version contains supplementary material available at 10.3758/s13423-021-01998-7.

Individual differences in behaviors are seen across many species (Araya-Ajoy & Dingemanse, 2014; Bell, 2007; Jandt et al., 2014; Matzel & Sauce, 2017; Reed & Pizzimenti, 1995). Individuals display different sets of correlated behaviors from one another that tend to co-occur across situations and contexts (Sih et al., 2004), and which may be related to underlying traits (Araya-Ajoy & Dingemanse, 2014; Matzel & Sauce, 2017). In the context of nonhumans, these types of individual differences have been referred to as ‘behavioral types’ (Araya-Ajoy & Dingemanse, 2014), or ‘behavioral syndromes’ (Bell, 2007; Sih et al., 2004). Investigations of such behavioral types or syndromes have focused on a variety of behavioral systems: emotionality (e.g., Flint et al., 1995; Hall, 1934); risk taking (Cremona et al., 2015; Araya-Ajoy & Dingemanse, 2014; Franks et al., 2014); boldness (Sinn et al., 2008); aggression (Cremona et al., 2015); and coping styles related to emotion and stress-provoking situations (Boakes et al., 2016; Coppens et al., 2010). Other research has focused on differences in cognitive systems that might underlay a range of behaviors (Coppens et al., 2010; Matzel & Sauce, 2017; Rayburn-Reeves et al., 2013; Reed & Pizzimenti, 1995; Sauce et al., 2014).

Many cognitive systems could be explored in terms of the existence of individual differences, but tasks potentially involving proactive interference have empirical, theoretical, and potentially clinical, importance. Proactive interference involves a degree of disengagement from a now-irrelevant stimulus in order to learn about a now-relevant stimulus. In the human learning literature, it is suggested that individual differences exist in proactive interference tasks (Dempster, 1985; Hedden & Yoon, 2006), and such considerations have been involved in the development of models of several disorders (Bartus et al., 1979; Hemsley, 2005; Morris et al., 2013; Verwoerd et al., 2009).

Several training procedures have received empirical attention in relation to individual differences, models of disorders, and/or proactive interference. In latent inhibition (LI), conditioning to a stimulus is retarded following repeated, nonreinforced presentations—the previously irrelevant stimulus becomes relevant in the conditioning stage (Lubow, 1989; Reed et al., 1999). The partial reinforcement extinction effect (PREE) refers to increased resistance to extinction of an operant response acquired under partial reinforcement relative to continuous reinforcement (Amsel, 1992; Bouton, 1993)—the stimulus in the acquisition phase sometimes becomes relevant (reinforcement), and sometimes irrelevant (no reinforcement), and in the extinction phase, the stimulus becomes continuously irrelevant. Reversal learning (RL) occurs when the target stimulus of the original training becomes the nontarget stimulus during the reversal training and vice versa (Mackintosh et al., 1968)—the initially relevant stimulus becomes irrelevant, while the previously irrelevant stimulus becomes relevant.

To the extent that individual differences in this cognitive system exist, then performance across these types of tasks may be expected to be similar for an individual. Empirically, data on whether such tasks relate to one another are mixed. Sauce et al. (2014) studied performance on four learning tasks and noted that LI and RL performances were associated. However, PREE was not found not to be related to LI by Yee, Feldon, and Rawlins (1997). Thus, while some reports have clearly found similarities in performance across proactive interference type tasks, others have not. As further exploration of the limits to individual differences in nonhumans is needed, putative proactive interference tasks provide a good opportunity to test this view.

Given this, the current study aimed to explore the relationship between performance across a range of proactive interference tasks by employing techniques from the ‘behavioral types’ literature—exploring the within-subject similarities in performance across tasks (Araya-Ajoy & Dingemanse, 2014; Matzel & Sauce, 2017). This presents an opportunity to develop a relatively new way of comparing nonhuman and human studies. To this end, the study explored common factors underlying proactive interference tasks, and whether there were clusters of subjects that demonstrated similar performance across all of the tasks.

## Method

### Subjects

Thirty-nine Lister Hooded rats, approximately 12 months old at the start of the study, were used. The animals were housed in groups of four, with water constantly available in the home cage. They had a free-feeding body weight range of 460–600 g, and were maintained at 85% of this weight throughout the experiments. All applicable international, national, and/or institutional guidelines for the care and use of animals were followed.

### Apparatus

#### Partial reinforcement extinction effect

Training was conducted in a straight alley made out of Perspex. The alley was nontransparent (black) on the sides with a transparent top. The runaway was 156-cm long, 20-cm wide, and 37-cm high, with a start-box (25-cm long), and a goal-box (25-cm long), separated by a 106-cm run section. The floor was the wooden surface of a long table. Both start-box and goal-box doors were made of nontransparent (grey) Perspex and opened vertically upwards. The doors were operated mechanically and were controlled by two push buttons. Reinforcement (five 45-mg food pellets) was placed manually, before each rewarded trial, on a food tray located at the end of the goal box. The food tray was covered by a clear, hinged flap. Running speed was measured manually by a stopwatch.

#### Latent inhibition task

Training was conducted in four identical operant-conditioning chambers (Camden Instruments Ltd.), from which the levers had been withdrawn. The chambers were ventilated by a fan, that provided a 68-dB(A) background noise. One 45-mg food pellet, delivered to a food tray covered by a clear, Perspex-hinged flap, served as reinforcement. A micro switch was operated when the flap was opened. A jeweled house-light was located on the center of the chamber ceiling. Another light was located centrally on the chamber wall above the food tray. Both lights employed 2.8-W bulbs. Based on past studies, both stimuli were of equal salience (Reed et al., 1999).

#### Reversal learning task

Four operant-conditioning chambers similar to those described for the LI task were used, into which the left lever had been inserted (the right remained withdrawn). The only visual stimuli that operated were the two 2.8-W bulbs, each located on the chamber wall above each lever.

### Procedure

#### Partial reinforcement extinction effect

The first task assessed the relative degree of resistance to extinction induced by partial reinforcement. Subjects were partially reinforced, and then were tested in extinction. This procedure was employed not to demonstrate the existence of a PREE, which is well documented, but to assess the relative degree to which it was induced across subjects. At the start of the experiment, the rats were given 3 days of pretraining. On Day 1 of pretraining, rats were introduced to the alley in groups of four for 20 min. All runway doors were open, and food pellets were spread all over the goal-box and the food tray. On Day 2, subjects were placed in the alley in pairs for 10 min, and, on Day 3, were placed individually in the runaway for 5 min (in both cases, food pellets were spread all over the goal-box and the food tray). On the fourth day of the study, the acquisition phase began. This phase consisted of 10 sessions (one session a day). On each session, every subject received two trials, with a 5–6-min intertrial interval. On each trial, the subject was placed in the start-box, and after 10 s, the start-box door was elevated simultaneously with the goal-box door. After reaching the food at the goal-box, the goal-box door was closed, and the rat was kept there for 10 s. Subjects were reinforced in a 50%, quasirandom schedule: Day 1-RR, Day 2-RN, Day 3-NN, Day 4-RN, Day 5-NR, Day 6-NR, Day 7-RR, Day 8-NR, Day 9-RN, Day 10-NR (where R is a rewarded trial, and N a nonrewarded trial: so, RR would be one rewarded trial followed by another rewarded trial; NR would be a nonrewarded trial followed by a rewarded trial, etc.). In the extinction phase (six two-trial sessions, one session per day), rats were treated as described in the acquisition phase, but no reward was given. Any subject failing to move from one section of the runaway to the other section within 20 s was removed from the apparatus and was given a score of 20 s for the trial.

#### Latent inhibition

The second task investigated the extent to which LI would be noted. Subjects were initially exposed to a stimulus (CS_PE_), and then were conditioned both to CS_PE_ and to another non-preexposed stimulus (CS_NPE_). The LI task took place two weeks after the PREE phase to allow baseline weights to be reestablished. Preexposure (Phase 1) consisted of eight 30-min sessions. In each session, the subjects received 10, 30-s unreinforced exposures to CS_PE._ Half of the subjects received an overhead light as CS_NPE,_ and a central light would serve as CS_PE_. The other rats received a central light as CS_NPE_, and an overhead light as a CS_PE_. The first stimulus presentation occurred 150 s after the onset of the session. All subsequent intertrial intervals were 150 s, as has been used in previous experiments using this procedure (Reed et al., 1999). Conditioning (Phase 2) consisted of six daily sessions. During each 30-min session, all subjects received 10, 30-s presentations of CS_PE_, immediately followed by reinforcement. In addition, they received 10, 30-s presentations of the stimulus CS_NPE_ followed, immediately after the offset, by reinforcement. The presentation of the two CSs was counterbalanced using a random, computer-generated order. Responses were entries into the magazine flap.

#### Reversal learning

The third task employed an instrumental visual reversal learning task (Reed et al., 1996). Subjects initially acquired a discrimination in which one stimulus (light or dark) signaled that responses would be reinforced, and the other stimulus signaled responses would not result in food. The above pattern of reinforcement was reversed in a second phase. This procedure was adopted in an attempt to distinguish the type of learning accruing in this phase (instrumental), from that which may have occurred previously (classical), by removing any contingency in which the rats had to physically approach the source of the stimulus as part of the training. Initial discrimination learning took place 2 weeks after the LI task to allow baseline weights to be reestablished. All subjects received light–dark discrimination training for 20 days. In each 10-min session, they received 15, 20-s light periods (the chamber was illuminated by two lights located above the levers—these were different lights from those employed in the LI task); and 15, 20-s dark periods (no light was operating). These periods alternated, and there was no ITI. During a light period (S+), for half the rats, a single lever press was reinforced by a food pellet. During a dark period (S−) for these rats, no reinforcement was given. The other half of the rats received food for pressing during the dark but not the light periods. The presentation of the dark and light periods was counterbalanced using a random, computer-generated order. The number of correct responses (lever pressing during the S+ period) was recorded. The reversal shift took place immediately after the end of the dark–light discrimination training, and reinforcement contingencies of the previous training were reversed. This training lasted for 23, 10-min sessions.

### Analysis

The first stage was to establish that the procedures produced the expected results—that is, there was extinction in the PREE; the CS_PE_ conditioned slower than the CS_NPE_ in LI; and discrimination learning occurred in both phases of the RL task, but more slowly in the second phase. Following this, data were examined to determine whether performances across the three tasks were related using an exploratory factor analysis with an unweighted means solution. This has been taken to be the optimal solution when sample sizes are relatively low (<30), and there are few expected factors (Jung, 2013). The current sample size for an expected maximum of 3 factors was adequate. Finally, to determine whether subjects could be classified in terms of their performance on the tasks, a cluster analysis was performed. There is no generally agreed rule for minimum sample size for cluster analysis (Siddiqui, 2013), but is has been suggested that 5*2^n^ (where n = clustered variables) would suffice; in this case this would be 40 participants (Formann, 1984), which the current sample approached closely.

As the tasks employed in this study all used different measures, it was necessary to calculate a common index to assess possible similarity in performance across tasks. This index reflected the rate of the individual’s performance for the new-target as a function of the previous target throughout each experimental task. In order to extract a limited number of representative variables for use in the subsequent factor analysis, the slope (β coefficient) of the regression line between each subject’s performance (dependent variable) and the session of training (predictor) was calculated. The β coefficient was preferred to the mean level of performance for two reasons: Firstly, the mean is sensitive to extreme values, which potentially can mask any existent covariance between the variables; secondly, the mean is expressed in terms of the units of the associated variable, thereby making cross-experimental comparisons less appropriate. In contrast, β coefficients use standardized data that can be directly compared across different indices (Hair et al., 1998).

To this end, three sets of linear regression analyses were conducted for each subject. On the mean extinction ratio (PREE), calculated as speed on current session divided by mean speed of the last session plus the mean speed on current session (to eliminate distortions in the interpretation of results stemming from differences in speed between different subjects). The smaller the slope value, the less quickly extinction occurred, and the stronger the PREE). On the difference between the mean elevation ratio between CS_NPE_ minus CS_PE_ (LI). The larger the value, the greater the LI. Finally, on the impact of previous training on the rate of RL expressed as the slope of the mean ratio of correct responses in Phase 1 minus that to the reversed target (RL) in Phase 2. A large positive difference between the slope values (Phase 1 minus Phase 2), reflects slower learning in the RL stage. Thus, high LI, and low PREE and RL, scores represent high proactive interference. This produced three variables for analysis, which although somewhat low is considered the practical minimum for a factor analysis (Tabachnick & Fidell, 2007; Velicer & Fava, 1998; Yong & Pearce, 2013).

## Results

**Fig. 1:**
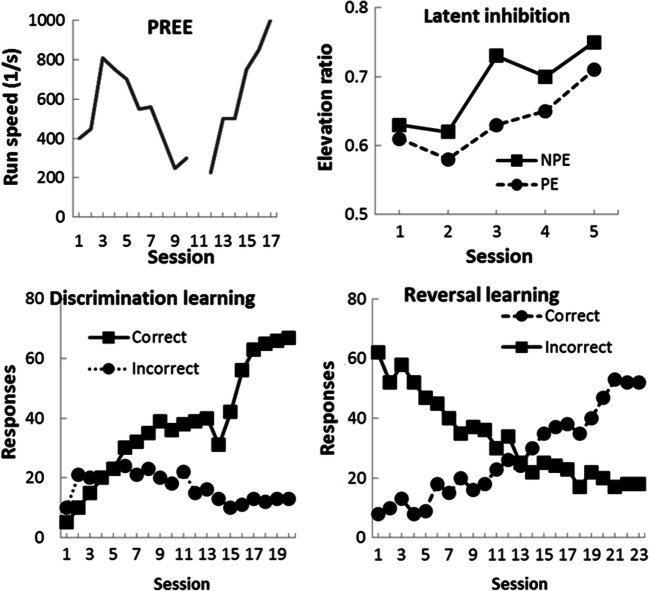
Top right = group-mean running speeds during acquisition and extinction phase expressed as two-trial, daily sessions. Top left = group-mean elevation ratio in five daily sessions for preexposed (PE) and non-preexposed stimulus (NPE). Bottom right = group-mean discrimination training ratios for correct responses. Bottom left = group-mean reversal learning ratios of correct responses

The top left panel of Fig. 1 presents the group-mean running speed during both acquisition and extinction phases of the PREE task. Inspection of these data reveals that, after an initial decrease, running speed gradually increased under the partial reinforcement training. It was noted that several of the repeated-measures data collected here violated the sphericity assumption according to Mauchly’s test, and, as a consequence, the Greenhouse and Geisser adjustment to degrees of freedom was adopted for all analyses, as recommended by Howell (1992). A repeated-measures analysis of variance (ANOVA), with session as a within-subject factor, was conducted on these data revealed a statistically significant effect, *F*(4.1, 342) = 20.60, *p* < .001, η_p_^2^ = .194, 95% CI [.118, .249], *pH*_*1*_*/D* = .759. Inspection of the extinction data revealed that the mean running speed gradually decreased across the daily sessions. A repeated-measures ANOVA, with session as a within-subject factor, revealed a significant effect of session, *F*(2.9, 34) = 32.20, *p* < .001, η_p_^2^ = .733, [.526, .806], *pH*_*1*_*/D* = .998.

The top right panel of Fig. 1 displays the group-mean elevation ratios for both stimuli across the six conditioning sessions of the LI task. The elevation ratio was calculated by measuring the total number of magazine entries made during the CS period, and dividing this number by the sum of entries made during the CS period and the entries made during the 30 s prior to a CS. There was a gradual increase in the elevation ratio for both CS_PE_ and CS_NPE_ across the sessions. However, conditioning to CS_PE_ was constantly lower than that of CS_NPE_. This description was supported by a two-factor repeated-measures ANOVA, with stimulus type (preexposed versus non-preexposed) and session as factors. The analysis revealed significant main effects of stimulus type, *F*(1, 38) = 15.10, η_p_^2^ = .284, [.069, .474], *pH*_*1*_*/D* = .991, and session, *F*(3.1, 119) = 15.60, *p*s < .001, η_p_^2^ = .289, [.147, .395], *pH*_*1*_*/D* = .771, but no significant interaction between stimulus type and session, *F* < 1, η_p_^2^ = .012, [.000, .052], *pH*_*0*_*/D* = .999.

The bottom left panel of Fig. 1 presents the group-mean ratio of correct and incorrect responses for the 20 sessions of discrimination learning training. The ratio of correct responses was calculated by dividing the number of correct responses per session by the total number of responses made during the session. Inspection of these data shows that, although subjects initially pressed the lever less often during the light periods, correct performance improved over training. A repeated-measures ANOVA conducted on the ratio of correct responses, with session as a factor, revealed a significant effect of session, *F*(7.68, 291.87) = 78.12, *p* < .001, η_p_^2^ = .682, [.620, .718], *pH*_*1*_*/D* = .999. The bottom right of Fig. 1 presents the mean ratio of correct responses for the 23 sessions of reversal learning. Subjects initially showed low levels of correct responding (during the dark periods). However, the overall rate of correct responding constantly increased across the sessions. A repeated-measures ANOVA conducted on the ratio of correct responses, with session as a factor, revealed a significant main effect of session, *F*(5.7, 210) = 55.30, *p* < .001, η_p_^2^ = .600, [.509, .603], *pH*_*1*_*/D* = .999.

### Exploratory factor analysis: Common factors across tasks

**Fig. 2:**
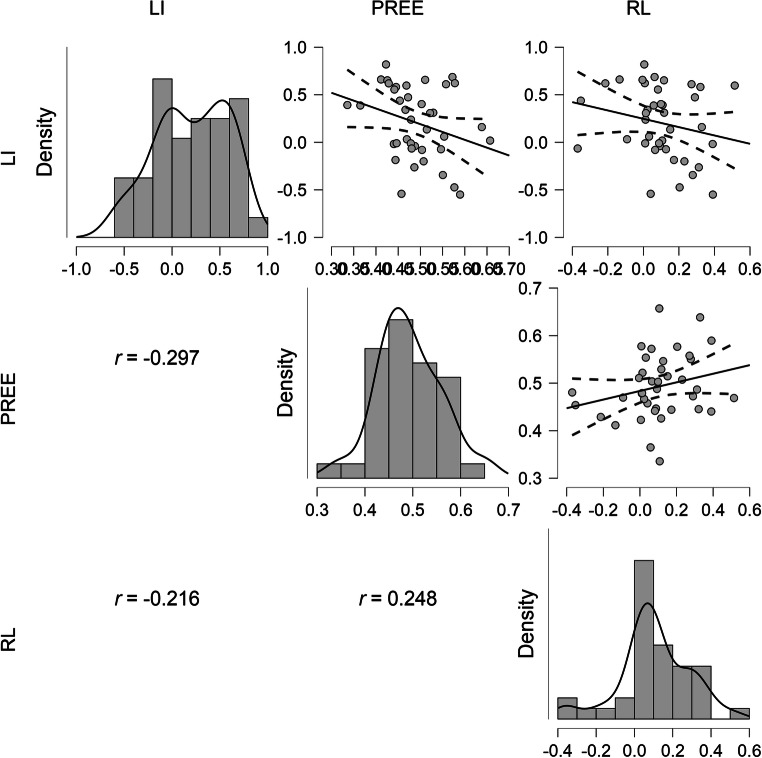
Correlations between each variable, histograms for each variable, and scatterplots with the regression line and 95% confidence intervals

Table 1 presents the sample means (standard deviations) and distribution statistics for each of the three tasks, along with their Pearson correlations. All tasks had acceptable levels of skewness and kurtosis, and Shapiro–Wilk tests found no significant deviations from normality. Figure 2 displays the correlations between each variable, histograms for each variable, and scatterplots with the regression line and 95% confidence intervals.

**Table 1:** Sample means (standard deviations) and distribution statistics for each of the three tasks, along with their Pearson correlations

	Mean (*SD*)	Skew	Kurt	Shapiro–Wilk	PREE	RL
LI	.222 (.360)	−.279	−.902	.953 (*p* = .113)	−.297	−.216
PREE	.490 (.067)	.312	.608	.094 (*p* = .534)		.248
RL	.101 (.185)	−.369	.919	.957 (*p* = 137)

To explore the existence of common components in the performance produced by the experimental tasks, the data were subjected to an exploratory factor analysis using the unweighted means solution. One factor was extracted, based on eigenvalues, scree plot, and also through running a parallel analysis that exceeded the 95% percentile (1,000 data sets). The results of these analyses are displayed in Table 2. The factor accounted for 50% of the variance. Inspection of the loadings revealed that the LI score loaded negatively, but the PREE and RL scores loaded positively. Indicating that the factor possibly reflected the operation of a single latent variable of not being impacted by proactive interference. To ensure that a one-factor solution did not result from any factor analysis conducted with this number of subjects and variables, a series of these factor analyses were performed, using the three proactive interference variables described here, and three acquisition variables drawn from these data. None of these analyses produced a clear one-factor solution, and these analyses are shown in detail in the supplementary materials.

**Table 2:** Results of exploratory factor analysis

Initial extraction					
Variable	Communality	Factor	Eigenvalue	% variance	Cumulative variance
LI	.261	1	1.509	50.293	50.29
PREE	.338	2	.792	26.416	76.71
RL	.191	3	.699	23.29	100.00
Unrotated factor matrix					
Variable	Factor 1				
LI	−.510				
PREE	.582				
RL	.425				

### Cluster analysis: Individual differences in performance

**Fig. 3:**
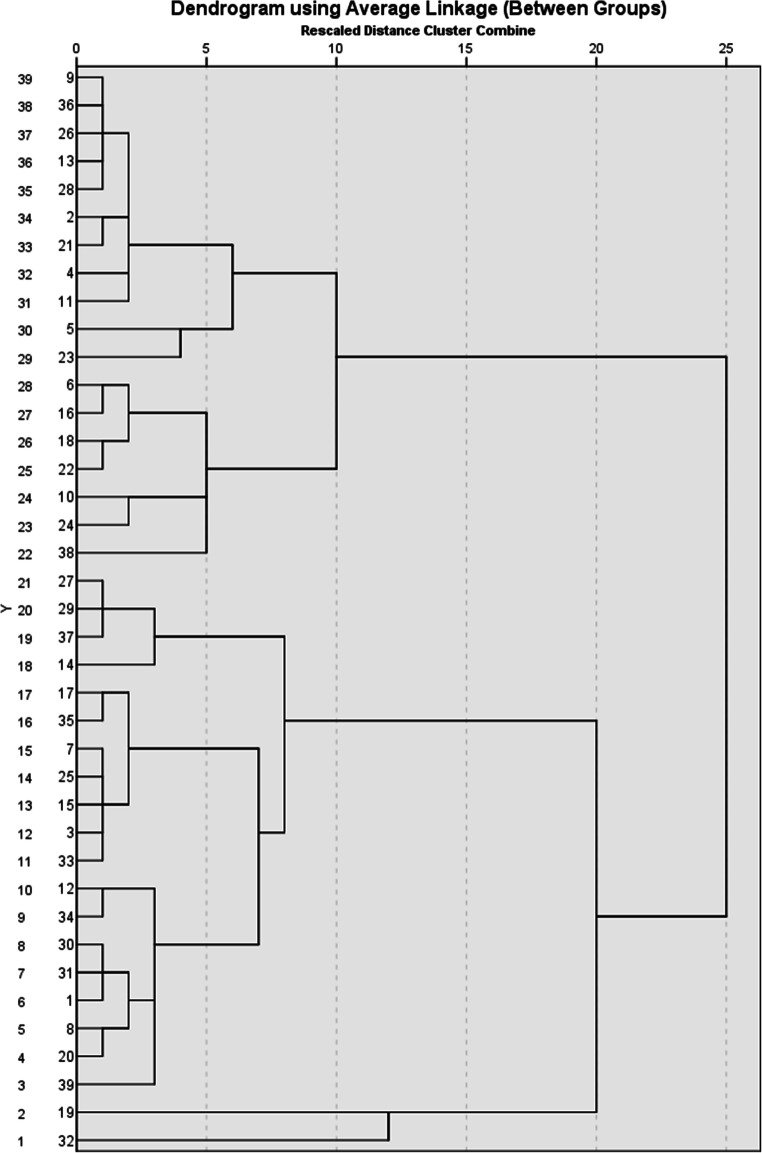
A dendrogram produced by the cluster analysis on the LI scores, based on an agglomeration schedule using the average linkage (within groups)

A cluster analysis was performed on the β values from the three tasks to reveal whether any set of subjects performed consistently well or poorly across these tasks. An agglomeration schedule using the average linkage within groups produced the Dendrogram of the cluster analysis displayed in Fig. 3. Subjects were assigned to clusters by using the clusters with the smallest average linkage (Euclidian distance). The dendrogram displays the results in such a way that, for example, Subject 9 can be seen to be most like Subject 36. Inspection of these data suggests that there were two coherent clusters capturing 95% (37/39) of the rats (all rescaled distances <10). The first cluster (*n* = 18), comprised Subject 9 down to 38; the second cluster (*n* = 19), Subject 27 down to 39; and Subjects 19 and 32 not fitting clearly into a cluster.

In order to evaluate the clusters, the mean scores of the subjects in each cluster for each task were calculated, and are shown in Table 3. From these data, it is apparent that Cluster 2 displayed more LI than Cluster 1, *t*(35) = 10.35, *p* < .001, *d* = 3.42. There was little difference between the clusters in terms of RL, *t*(35) = 1.41, *p* = .166, *d* = .48. Clusters 2 showed greater PREE than Cluster 1, *t*(35) = 2.28, *p* = .029, *d* = .50.

**Table 3:** Mean scores on each task for the three clusters

Cluster	Subjects	LI	RL	PREE
1	6–38	−.132 (.221)	.162 (.137)	.519 (.060)
2	27–39	.522 (.161)	.092 (.165)	.470 (.066)

*Note. RL* reversal learning, *PREE* partial reinforcement extinction effect, *LI* latent inhibition. For further explanation, see text.

## Discussion

The current study investigated whether there were any consistent individual differences in rats’ performance (behavioral traits/syndromes) across tasks that involve a degree of proactive interference. That the obtained factor structure contained one factor, with greater amounts of LI, a stronger PREE, and retarded RL, loading onto the factor. This supports the suggestion that performance across these tasks reflects the operation of a single system—all of these effects being consistent with displaying stronger proactive interference. This extends the range of potential structures on which nonhumans may show consistent individual differences similar to that noted in humans (Dempster, 1985; Hedden & Yoon, 2006). Previous studies have noted such behavioural types in terms of emotionality (Flint et al., 1995), risk taking (Araya-Ajoy & Dingemanse, 2014), boldness (Sinn et al., 2008), and aggression (Cremona et al., 2015). Other research has focused on differences in cognitive systems, such as attention (Matzel & Sauce, 2017; Sauce et al., 2014) and flexibility (Rayburn-Reeves et al., 2013), but not inhibition (Reed & Pizzimenti, 1995). Thus, these data add to the limited available evidence for rats relating to individual differences in cognitive systems (e.g., Reed & Pizzimenti, 1995; Rayburn-Reeves et al., 2013; Sauce et al., 2014).

One feature of the current results that is worth brief comment is that LI and PREE performance loaded most strongly to this factor, with RL being less strongly associated. Whereas both LI (Lubow, 1989; Reed et al., 1999) and PREE (Amsel, 1992; Bouton, 1993) have been discussed often in terms of proactive interference, as has RL (Mackintosh et al., 1968), this explanation for RL has been disputed (Calhoun & Handley, 1973). Whatever the eventual resolution of this particular debate, the current method may offer opportunities to provide evidence of the operation of particular processes in novel tasks. For any given task, to the degree that it shares in a particular operation, then it should load onto a factor containing procedures that are known to share in that operation.

In terms of the individual differences across the tasks noted in the current study, the cluster analysis revealed a set of subjects (Cluster 1) demonstrating substantially reduced LI and PREE, along with slightly faster RL. All of these results indicating a weaker influence of proactive interference. This result is potentially interesting, as disruption of LI has been taken as a starting point for the development of an animal model of schizophrenia (Hemsley, 2005). The current results allowed examination of whether a naturally occurring cluster of subjects that showed only small LI, would demonstrate differential performance in the rest of the experimental tasks (many of which have also been shown to be disrupted in schizophrenia). Of particular importance in this regard was the performance on the PREE and LI tasks, which have been heavily used in the development of models of schizophrenia (Clark et al., 1992).

More broadly in the context of nonhuman behavioral syndromes/traits (Bell, 2007; Araya-Ajoy & Dingemanse, 2014; Matzel & Sauce, 2017) studies have only produced limited evidence that nonhuman behavioral traits/syndromes can be related to an underlying cognitive system (cf. Reed & Pizzimenti, 1995; Rayburn-Reeves et al., 2013; Sauce et al., 2014). This stands in contrast to the growing evidence that there are such individual differences when the system being explored might be characterized as behavioral (e.g., boldness, aggression, risk taking as opposed to attention). Clearly, there is much conceptual work to be undertaken in distinguishing between these concepts, which is beyond the scope of this study. Moreover, the relationship of the individual components of a human personality trait (cognition, behavior, affect) will need further exploration in the context of nonhuman traits/syndromes. However, the current study offers further evidence of the existence of such cognitive behavioural traits/syndromes, as well as potential techniques to investigate the underlying processes of novel tasks.

## Supplementary Information

ESM 1 (DOCX 170 kb)
