# Supplementary material for: Individual differences in proactive interference in rats (Rattus Norvegicus)
Source: Psychon Bull Rev. 2021 Sep 24;29(1):203–11. doi: 10.3758/s13423-021-01998-7 (PMC8858310; doi:10.3758/s13423-021-01998-7)
Supplement: Supplementary file 1 — (DOCX 170 kb) [file 13423_2021_1998_MOESM1_ESM.docx]

**Supplementary materials**

To explore whether one factor would be generated no matter which variables were employed from this study, a series of factor analyses were performed using the three proactive interference (PI) variables, and three acquisition (A) variables. One was calculated from the regression slopes for the non-preexposed stimulus in the latent inhibition (LI) task; one from the rate of acquisition in the runway during partial reinforcement extinction effect training (PREE) and one from the initial discrimination learning part of the reversal learning task (RL). None of these analyses produced a clear one factor solution.

Figure S1 displays the correlations between each acquisition variable, histograms for each variable, and scatterplots with the regression line and 95% confidence intervals.

**Fig. S1** Correlations between each acquisition variable, histograms for each variable, and scatterplots with the regression line and 95% confidence intervals


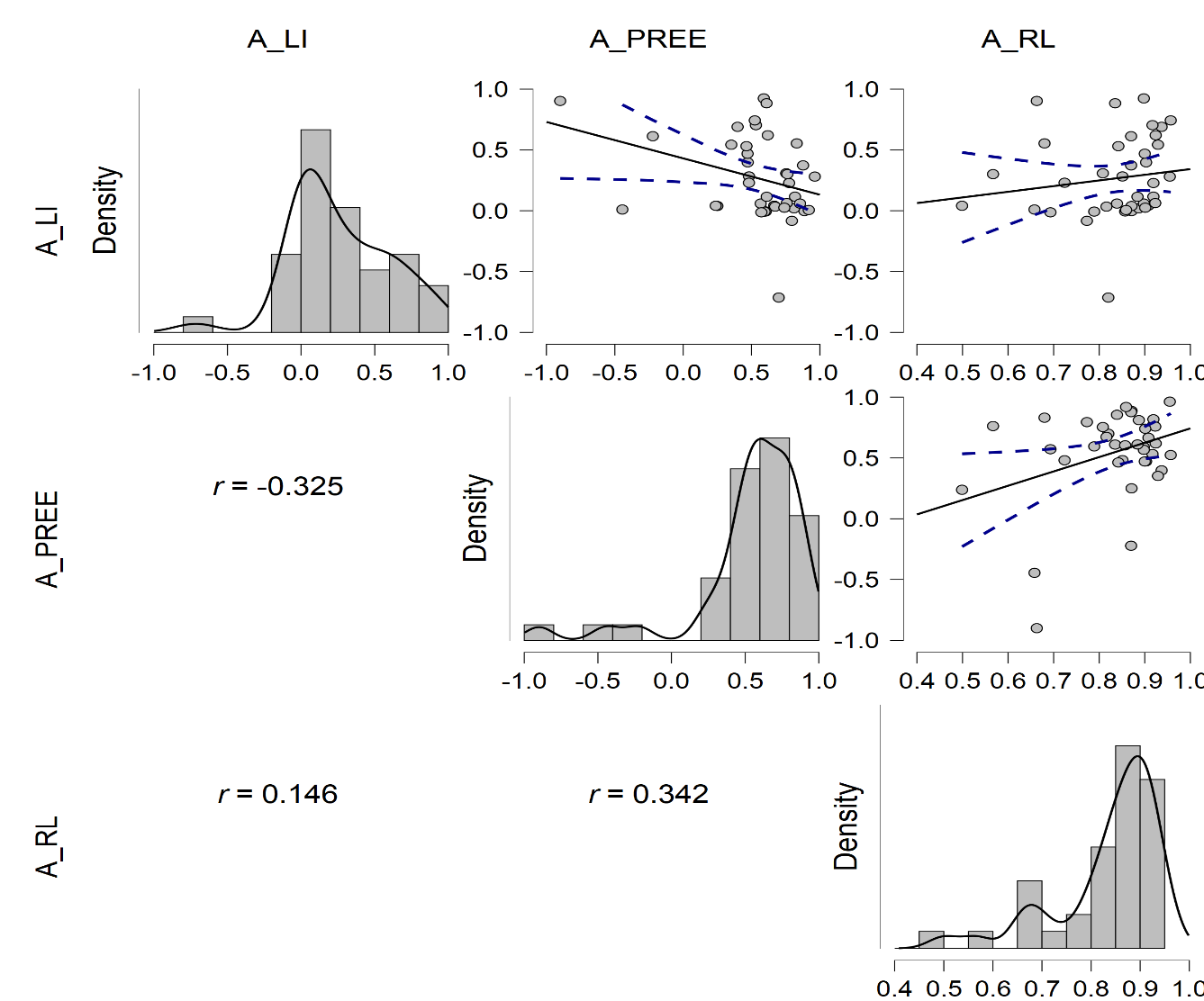


**Table S1** Results of exploratory factor analysis conducted on acquisition variables

Initial extraction

**----------------------------------------------------------------------------------------------------------------**

Variable Communality Factor Eigenvalue % variance Cumulative

variance

**----------------------------------------------------------------------------------------------------------------**

A_LI .180 1 1.404 46.793 46.79

A_PREE .260 2 1.146 38.209 94.99

A_RL .191 3 .450

----------------------------------------------------------------------------------------------------------------

Unrotated factor matrix

-----------------------------------------------------------

Variable Factor 1 Factor 2

-----------------------------------------------------------

A_LI −.515 .780

A_PREE .899 −.021

A_RL .425 .733

------------------------------------------------------------

To explore the existence of common components in the performance produced by the experimental tasks, the data were subjected to an exploratory factor analysis using the unweighted means solution. Two factors were extracted, based on eigenvalues, scree plot, and also through running a parallel analysis that exceeded the 95% percentile (1,000 data sets). The results of these analyses are displayed in Table S1. The two factors accounted for 94% of the variance. Inspection of the loadings revealed that A_PREE loaded onto one factor, and A_LI and A_RL loaded onto the other.

**Table S2** Results of exploratory factor analysis conducted on LI acquisition, and PREE and RL proactive inference, variables

Initial extraction

**----------------------------------------------------------------------------------------------------------------**

Variable Communality Factor Eigenvalue % variance Cumulative

variance

**----------------------------------------------------------------------------------------------------------------**

A_LI .933 1 1.285 42.171 42.171

PI_PREE .845 2 1.010 33.674 75.84

PI_RL .888 3 .725

----------------------------------------------------------------------------------------------------------------

Unrotated factor matrix

-----------------------------------------------------------

Variable Factor 1 Factor 2

-----------------------------------------------------------

A_LI .294 .923

PI_PREE .802 .036

PI_RL .735 −.396

------------------------------------------------------------

To explore the existence of common components in the performance produced by these variables, an exploratory factor analysis using the unweighted means solution. Two factors were extracted, based on eigenvalues, scree plot, and also through running a parallel analysis that exceeded the 95% percentile (1,000 data sets). The results of these analyses are displayed in Table S2. The two factors accounted for 75% of the variance. Inspection of the loadings revealed that A_LI loaded onto one factor, and PI_PREE and PI_RL loaded onto the other. Indicating that the factors possibly reflected the operation of two variables, most likely acquisition and proactive interference.

**Table S3** Results of exploratory factor analysis conducted on PREE acquisition, and LI and RL proactive inference, variables

Initial extraction

**----------------------------------------------------------------------------------------------------------------**

Variable Communality Factor Eigenvalue % variance Cumulative

variance

**----------------------------------------------------------------------------------------------------------------**

PI_LI .803 1 1.286 42.883 42.883

A_PREE .840 2 1.007 33.561 76.445

PI_RL .851 3 .707

----------------------------------------------------------------------------------------------------------------

Unrotated factor matrix

-----------------------------------------------------------

Variable Factor 1 Factor 2

-----------------------------------------------------------

PI_LI −.595 .670

A_PREE .530 .747

PI_RL .807 .003

------------------------------------------------------------

Rotated factor matrix (varimax)

-----------------------------------------------------------

Variable Factor 1 Factor 2

-----------------------------------------------------------

PI_LI .885 .142

A_PREE .062 .914

PI_RL −.623 .513

------------------------------------------------------------

To explore the existence of common components in the performance produced by these variables, an exploratory factor analysis using the unweighted means solution. Two factors were extracted, as described above. The results of these analyses are displayed in Table S3. The two factors accounted for 76% of the variance. Inspection of the unrotated loadings revealed an unclear structure, but a varimax rotation suggested that PI_LI loaded onto one factor, and A_PREE and PI_RL loaded onto the other.

**Table S4** Results of exploratory factor analysis conducted on RL acquisition, and LI and PREE proactive inference, variable

Initial extraction

**----------------------------------------------------------------------------------------------------------------**

Variable Communality Factor Eigenvalue % variance Cumulative

variance

**----------------------------------------------------------------------------------------------------------------**

PI_LI .660 1 1.297 43.339 43.339

PI_PREE .658 2 1.006 33.534 76.773

A_RL .986 3 .597

----------------------------------------------------------------------------------------------------------------

Unrotated factor matrix

-----------------------------------------------------------

Variable Factor 1 Factor 2

-----------------------------------------------------------

PI_LI .804 −.115

PI_PREE −.808 −.085

A_RL .024 .993

------------------------------------------------------------

To explore the existence of common components in the performance produced by these variables, an exploratory factor analysis using the unweighted means solution. Two factors were extracted. Two factors were extracted, based on eigenvalues, scree plot, and also through running a parallel analysis that exceeded the 95% percentile (1,000 data sets). The results of these analyses are displayed in Table S4. The two factors accounted for 76% of the variance. Inspection of the unrotated loadings revealed that PI_LI and PI_PREE loaded onto one factor, and A_RL loaded onto the other—most likely reflecting acquisition and proactive interference influences.

**Table S5** Results of exploratory factor analysis conducted on LI and PREE acquisition, and RL proactive inference, variables

Initial extraction

**----------------------------------------------------------------------------------------------------------------**

Variable Communality Factor Eigenvalue % variance Cumulative

variance

**----------------------------------------------------------------------------------------------------------------**

A_LI .509 1 1.384 37.868 37.868

A_PREE .891 2 .988

PI_RL .195 3 .628

----------------------------------------------------------------------------------------------------------------

Unrotated factor matrix

-----------------------------------------

Variable Factor 1

-----------------------------------------

A_LI −.713

A_PREE −.825

PI_RL .442

------------------------------------------

To explore the existence of common components in the performance produced by these variables, an exploratory factor analysis using the unweighted means solution. One factor was extracted based on Eigenvalues, Scree plot, and also through running a parallel analysis that exceeded the 95% percentile (1,000 data sets), but only accounted for 37% of these data. The results of these analyses are displayed in Table S5.

**Table S6** Results of exploratory factor analysis conducted on LI and RL acquisition, and PREE proactive inference, variables

Initial extraction

**----------------------------------------------------------------------------------------------------------------**

Variable Communality Factor Eigenvalue % variance Cumulative

variance

**----------------------------------------------------------------------------------------------------------------**

A_LI .637 1 1.116 38.857 38.857

PI_PREE .828 2 1.033 34.437 73.293

A_RL .733 3 .801

----------------------------------------------------------------------------------------------------------------

Unrotated factor matrix

-----------------------------------------------------------

Variable Factor 1 Factor 2

-----------------------------------------------------------

A_LI .786 .064

PI_PREE .383 .828

A_RL .622 .588

------------------------------------------------------------

Rotated factor matrix (varimax)

-----------------------------------------------------------

Variable Factor 1 Factor 2

-----------------------------------------------------------

A_LI .679 .416

PI_PREE −.037 .909

A_RL .822 −.240

------------------------------------------------------------

To explore the existence of common components in the performance produced by these variables, an exploratory factor analysis using the unweighted means solution. Two factors were extracted, as described above, that accounted for 73% of these data. The results of these analyses are displayed in Table S6, but are somewhat unclear in both the unrotated and rotated analyses.

**Table S7** Results of Exploratory Factor Analysis conducted on LI proactive interference, and PREE and RL acquisition, variables

Initial extraction

**----------------------------------------------------------------------------------------------------------------**

Variable Communality Factor Eigenvalue % variance Cumulative

variance

**----------------------------------------------------------------------------------------------------------------**

PI_LI .997 1 1.342 44.736 44.736

A_PREE .874 2 1.001 33.388 78.104

A_RL .657 3 .857

----------------------------------------------------------------------------------------------------------------

Unrotated factor matrix

-----------------------------------------------------------

Variable Factor 1 Factor 2

-----------------------------------------------------------

PI_LI −.046 .997

A_PREE .817 .078

A_RL .820 −.020

------------------------------------------------------------

To explore the existence of common components in the performance produced by these variables, an exploratory factor analysis using the unweighted means solution. Two factors were extracted, as described above, that accounted for 78% of these data. The results of these analyses are displayed in Table S7, and suggest the LI proactive interference variable loaded onto one factor, and the two acquisition variables loaded onto the second variable.
